# Singularity: Scientific containers for mobility of compute

**DOI:** 10.1371/journal.pone.0177459

**Published:** 2017-05-11

**Authors:** Gregory M. Kurtzer, Vanessa Sochat, Michael W. Bauer

**Affiliations:** 1 High Performance Computing Services, Lawrence Berkeley National Lab, Berkeley, CA, United States of America; 2 Stanford Research Computing Center and School of Medicine, Stanford University, Stanford, CA, United States of America; 3 Department of Electrical Engineering and Computer Science, University of Michigan, Ann Arbor, MI, United States of America; 4 Experimental Systems, GSI Helmholtzzentrum für Schwerionenforschung, Darmstadt, Germany; Koc Universitesi, TURKEY

## Abstract

Here we present Singularity, software developed to bring containers and reproducibility to scientific computing. Using Singularity containers, developers can work in reproducible environments of their choosing and design, and these complete environments can easily be copied and executed on other platforms. Singularity is an open source initiative that harnesses the expertise of system and software engineers and researchers alike, and integrates seamlessly into common workflows for both of these groups. As its primary use case, Singularity brings mobility of computing to both users and HPC centers, providing a secure means to capture and distribute software and compute environments. This ability to create and deploy reproducible environments across these centers, a previously unmet need, makes Singularity a game changing development for computational science.

## Introduction

The landscape of scientific computing is fluid. Over the past decade and a half, virtualization has gone from an engineering toy to a global infrastructure necessity, and the evolution of related technologies has thus flourished. The currency of files and folders has changed to applications and operating systems. The business of Supercomputing Centers has been to offer scalable computational resources to a set of users associated with an institution or group [1]. With this scale came the challenge of version control to provide users with not just up-to-date software, but multiple versions of it. Software modules [2, 3], virtual environments [4, 5], along with intelligently organized file systems [6] and permissions [7] were essential developments to give users control and reproducibility of work. On the administrative side, automated builds and server configuration [8, 9] have made maintenance of these large high-performance computing (HPC) clusters possible. Job schedulers such as SLURM [10] or SGE [11] are the metaphorical governors to control these custom analyses at scale, and are the primary means of relay between administrators and users. The user requires access to consume resources, and the administrator wants to make sure that the user has the tools and support to make the most efficient use of them.

The early days of these centers carried an atmosphere of friendly competition. Centers worked hard to offer larger and faster clusters, and to support different kinds and scales of work. As these centers grew, an emphasis on management, standards, stability, and service-level requirements became increasingly important. From a provider’s perspective, it was necessary to build a reliable and usable resource. From some researchers’ perspectives, however, this same reliability translated to old, stale, and immobile software and systems. This static nature coupled with distribution-specific software builds meant that service providers would ultimately end up limiting the scope of computational science that their systems could support, leaving out the long tails of science [12–14] and scientific diversity of nontraditional HPC user bases.

### Portable environments

The advent of virtual machines [4, 5] introduced the exciting reality than an entire environment, including software dependencies, libraries, runtime code, and data, could be encapsulated and run anywhere. Virtual machines, however, also introduced large computational overhead due to the required level of virtualization for emulating the OS and kernel. With the addition of lightweight virtualization features to the Linux kernel (e.g., namespaces) a new lightweight virtualization, containers [15, 16], became possible to implement. Implementations such as Docker, one of the container solutions made open source in 2013 [15, 16], offered additional improvements over standard virtual machines. Containers could share resources with the host without incurring much of the performance penalties of hardware-level virtualization [17].

The advent of containers has come in parallel with increased demand for customization by the user, meaning user-defined software stacks (UDSS) that require custom libraries, build environments, and commonly, entire operating systems. Portable environments address this need comprehensively. In less than a few decades, the early sharing of unreproducible installations of libraries and applications [13] has been extended to a need to share entire operating systems, especially for niche communities wanting to distribute and run UDSS.

#### Software unfit for scaled science

The promise of portable environments was not delivered quickly to all communities alike. For the industry at the forefront of virtualization, the implementation and feature set offered by container technologies is very much in alignment with their need for enterprise micro-service virtualization and web-enabled cloud applications. For the scientific world, and specifically HPC communities, the same technology does not fit cleanly. The reproducibility crisis [18–20] in early 2015 made container-based applications especially yearned for by these research communities, for which long term success hinges on the replication of both scientific findings and computational environments. For these groups, installing Docker on some kind of HPC environment would mean an unreasonable level of security risk. Thus, it was quickly apparent that these two general communities, despite having common features, differed in ways that would make a shared implementation generally incompatible. The unfortunate reality was that Docker could not be embraced by a large group of people that needed it greatly.

The lack of compatibility in cluster environments and computational centers did not prevent researchers from embracing containers for science. Several groups across genetics [21, 22], neuroscience [23], and others [24, 25] introduced container-based scientific analyses, and this was a reflection of two things. First, the growing popularity of large data did not warrant smaller data extinct or lacking value [26–28], and many researchers could thus develop reproducible containers on their local machines. Docker was well suited for this task, and it eliminated many headaches in providing a simple way to collaborate on code or applications without the hassle of having different software versions or broken dependencies. Second, containers were ideal not just for the final analysis, but for the development of it. A user is arguably most comfortable working with his or her text editor, programs, and environment of choice, and containers made it possible to work locally and develop in a specific environment simultaneously. While recent improvements have been made in extending a cluster resource to a user’s desktop (e.g. Jupyter Hub [29]), the most common cluster experience is still logging in to a machine via a command line with a secure shell (SSH), and needing to install software and scripts. If an internet connection is not available, this workflow may not even be possible. When comparing this headless option as a base requirement for working to an instantly available, local machine, the easier of the two is obvious. However, this mobility of compute afforded in a local environment is an entirely different problem than the need to scale computation.

#### Early initiatives for scientific containers

As researchers also started embracing containers for science, the desire to use these same containers at scale grew as well. HPC environments provided by research institutions began receiving requests to allow researchers to run their containers at scale. The community responded with a flood of efforts toward this goal, notably from national labs including Los Alamos National Lab (LANL) with CharlieCloud [30], the National Energy Research Scientific Computing (NERSC) with Shifter [31], and others risking or not caring about the security implications of Docker. While each solution has optimal use cases, each also comes with caveats and loopholes, discussed in more detail later in this paper.

Another growing initiative of many institutions has been, with careful work to address security and affordability, a movement to using cloud resources. In fact, behind the scenes of many institutions that do not have a formal relationship with a cloud provider, Principal Investigators (PIs) have pursued using cloud resources to move their research projects forward, including many notable individual efforts [32], some of which have driven the establishment of new standards and relationships [33, 34]. Ideally, the products developed by researchers across academia and industry should be agnostic to the deployment environment, whether that be a protected cluster, or an industry cloud provider such as Google Cloud Platform [35], Amazon Web Services [36], or Microsoft Azure [37]. Portability is essential for replication of the work, and so any product that is limited to where it can be deployed is instantly limited in the extent that it can be reproduced.

### The needs of scientists

The technological innovation of container-based environments, the need for scalable and reproducible products, a preference for usability, and the necessity to interoperate on everything from laptops to large scale HPC resources defines our current landscape. Data files and random bits of source code, the formats that were previously the currency of interest when exchanging scientific workflows, were replaced with software to manipulate them, and now the currency of interest is combining these two things into a single encapsulated unit. For the end-user, the lines are blurred between cloud and local compute environments: both are machines that the user must connect to that offer some amount of memory and disk space. Regardless, a container solution is badly needed that is agnostic to these details, and can move seamlessly between the two.

#### Singularity containers

Here we introduce Singularity, a container solution created by necessity for scientific application driven workloads. Singularity offers mobility of compute by enabling environments to be completely portable via a single image file, and is designed with the features necessary to allow seamless integration with any scientific computational resources. We will first discuss the problems and use cases that Singularity is ideal for, followed by talking about the software itself. Singularity is the first of its kind to be easy for both users and administrators, and was developed in collaboration by HPC administrators, developers and research scientists alike. We started our work aiming to solve the problem of a lack of reproducible, portable environments for science. Our hypothesis was that we could invent data structures and accompanying methods in a software package to make this possible. We have validated our work in the most powerful manner possible—installation and extensive use after detailed review by supercomputer centers globally. Singularity represents a direct implementation to demonstrate the validity of this novel scientific idea. We will focus our discussion on solving these problems and usage, and it will be followed by a comparison of Singularity to other Linux containers that do not address the problem fully. Finally, we will provide forward thinking about how Singularity fits in with this continually changing landscape.

## The problems that singularity solves

This section will briefly overview a selection of problems that Singularity aims to solve. We start with a comparison to currently existing solutions, followed by how Singularity addresses concerns with these approaches, and then example use cases of such problems.

### Available container solutions

While several software management solutions exist that provide flexibility and customization, including environment modules [3] for Linux environments in production (CHOS) [38], our focus will be on container solutions that house not just modular software, but potentially entire systems. We provide an overview of our comparison of the leading container technologies in (Table 1), which has been extended and adopted from [30]. As it is the case that technologies change quickly, the authors would like to note that this table, and the following discussion, is relevant to the time of writing of this paper.

**Table 1:** Container comparison.

	Singularity	Shifter	Charlie Cloud	Docker
Privilege model	SUID/UserNS	SUID	UserNS	Root Daemon
Supports current production Linux distros	Yes	Yes	No	No
Internal image build/bootstrap	Yes	No*	No*	No***
No privileged or trusted daemons	Yes	Yes	Yes	No
No additional network configurations	Yes	Yes	Yes	No
No additional hardware	Yes	Maybe	Yes	Maybe
Access to host filesystem	Yes	Yes	Yes	Yes**
Native support for GPU	Yes	No	No	No
Native support for InfiniBand	Yes	Yes	Yes	Yes
Native support for MPI	Yes	Yes	Yes	Yes
Works with all schedulers	Yes	No	Yes	No
Designed for general scientific use cases	Yes	Yes	No	No
Contained environment has correct perms	Yes	Yes	No	Yes
Containers are portable, unmodified by use	Yes	No	No	No
Trivial HPC install (one package, zero conf)	Yes	No	Yes	Yes
Admins can control and limit capabilities	Yes	Yes	No	No

In addition to the default Singularity container image, a standard file, Singularity supports numerous other formats described in the table. For each format (except directory) the suffix is necessary for Singularity to identify the image type.

*relies on Docker

**with security implications

***depends on upstream

#### Software modules and package managers

Environment modules [3] allow users to enable or disable software package by way of a user interface to manipulate paths to executables that can be found, and package managers ([39], [40]) perform installation to a location given that the user has write permission to that location. Modules and package managers make installation easy, however they are not portable environments in that they can encapsulate a workflow, data, and the entire operating system itself.

As an example, we can compare Singularity to the Nix package manager [40]. Nix supports reproducibility in that it can be installed across a range of hosts, and installs software to the host without conflicting with the host. However, a package manager is not a portable environments or a containerized workflow. Given that a user has set up a specific configuration of software packages, environment variables, and custom script for his or her analysis, as a package manager, Nix does not provide an easy means to package that entire environment (software, environment, and importantly, the operating system itself) to be moved and run anywhere. While an equivalent modular software might be installed on two different hosts, it would still not be possible to run an Ubuntu OS on CentOS, for example. In contrast, a container built with the Singularity software will include all of the environment variables, software, custom scripts, and operating system that the user specified, and can be moved seamlessly to another host and be executed. There is no dependency on needing to re-use some central package manager to install software, or even re-build the image at all. For these reasons, Singularity is fundamentally different than a package manager.

In summary, while package managers and modules are a cornerstone to traditional HPC, and are installed on almost every scientific center, they do not address the same problems as Singularity. For users that have preference for a package manager or module manager, however, Singularity would allow for installation of it in a container, and then immediate use on any cluster, even if the cluster does not natively support it.

#### Virtual machines

A discussion of containers would not be complete without a mention of virtual machines. Virtual machines [4, 5] (VM) are emulators for computer systems, and costly to run in that they deploy a layer of resource emulation and an entire duplicate operating system on top of the emulation layer. In the context of portable and reproducible environments, a user might create a specification for a VM that includes a download of dependencies, and installation of software. The virtual machine provides complete isolation, making it safe for users to obtain root privileges within that single environment, however this also could be a point of bypassing network and file system security [41–43]. Isolation also creates additional complexities when trying to access host specific resources such as scaled networking (e.g. InfiniBand [44]) or hardware accelerators. Finally, virtual machines are not quick to deploy or transfer between systems, and for the common scientist, this simple fact that usability is slow makes them less desirable to use.

#### CharlieCloud

Charliecloud is an open-source software based on the idea of user-defined software stack (UDSS). The original release notes [30] describe the software as “an industry-standard, reproducible workflow based on Docker” with an emphasis on being a user namespace implementation that removes the need for the user to have root privileges. The workflow of this software is to build a UDSS using Docker, extract (or untar) the contents into a controlled directory location, and then execute code within the directory by way of a C executable. CharlieCloud can be then be used for running Docker containers.

There are several prominent issues with this approach, the first being compatibility. The software makes use of kernel namespaces that are not deemed stable by multiple prominent distributions of Linux (e.g. no versions of Red Hat Enterprise Linux or compatibles support it), and may not be included in these distributions for the foreseeable future. A closely related issue is one of dependencies. The workflow begins with Docker. While Docker is becoming a standard technology in the enterprise industry, it would be desirable for this additional software to not require it for baseline operation. The software is emphasized for its simplicity and being less than 500 lines of code, and this is an indication of having a lack of user-driven features. The containers are not truly portable because they must be extracted from Docker and configured by an external C executable before running [30], and even after this step, all file ownership and permissions are dependent on the user running the workflow. Thus, while CharlieCloud is a good effort and still holds promise to develop further, due to its limited functionality, portability, and dependencies, it is not a robust mechanism for reproducibility.

#### Shifter

Shifter [31] is an effort led by NERSC, and was originally based on chroot(2) [45]. It also uses Docker as a base image building workflow. While it has been successfully shown to operate well for production workflows [45], the user must submit a finished image to a root controlled gateway, a Dockerized RESTful interface to handle creation, tracking, and management of images, to finish configuration. It connects seamlessly to host-based resources by way of bind mounts and integrates with the resource manager to provide a unified container-based workflow. This integration requires a complex administrative setup of the resource manager, daemons, and depending on the architecture needs, a complete hardware specific resource for the image gateway. Overall, like CharlieCloud, it is designed to work with (but modify) Docker containers and its setup and management is a non-trivial task.

#### Docker

As alluded to above, Docker is the industry standard for micro-service virtualization and it is a fantastic solution for this need, but unfortunately does not meet the requirements for widespread scientific computational usage quite as well. Docker caters to a specific need to package and deploy applications and services. It was the first software to provide an intuitive API to harness kernel-level technologies (e.g., linux containers, control groups, and a copy-on-write filesystems), and use namespaces (e.g. user, process, and network) to allow for isolation of containers from the host [46].

One of the major factors that prevents Docker from being the standard container technology in HPC is its security concerns. From an IT security perspective, a machine can be considered compromised if any user is able to run arbitrary code as the root user. While Docker takes steps to mitigate the risk of allowing users to run arbitrary code, there is a fatal design flaw that limits Docker’s ability to run in HPC environments: for every container that Docker runs, the container process is spawned as a child of a root owned Docker daemon. As the user is able to directly interact with and control the Docker daemon, it is theoretically possible to coerce the daemon process into granting the users escalated privileges. Any user being able to escalate up to system administrator status, a user called “root”, would introduce unthinkable security risks for a shared compute environment.

While this is not a problem for enterprise usage of Docker, as the system administrator knows what code will be running and the command being used to invoke Docker, a system administrator of an HPC center is not afforded the same level of control over what code is being run. One of the core challenges of running an HPC center dedicated to research is allowing users to run arbitrary code while simultaneously ensuring that the system is not compromised by malicious code (whether intentionally malicious or not).

### The goals of singularity

Having discussed the problem space and currently available solutions, we will next discuss how the Singularity can and has provided a robust solution to these issues.

#### Mobility of compute

Mobility of compute is defined as the ability to define, create, and maintain a workflow locally while remaining confident that the workflow can be executed on different hosts, Linux operating systems, and/or cloud service providers. In essence, mobility of compute means being able to contain the entire software stack, from data files up through the library stack, and reliability move it from system to system. Mobility of compute is an essential building block for reproducible science, and consistent and continuous deployment of applications.

Singularity achieves this by utilizing a distributable image format that encapsulates the entire container and stack into a single image file. This file can be copied, shared, archived, and thus all standard UNIX file permissions also apply. Additionally, Singularity containers are portable across different C library versions and kernel implementations.

#### Reproducibility

As mentioned above, Singularity containers utilize a single file which is the complete representation of all the files within the container. Many of the same features that facilitate mobility also facilitate reproducibility. Once a contained workflow has been defined, the container image can be snapshotted, archived, and locked down such that it can be used later and the user can be confident that the code within the container has not changed. The container is not subject to any external influence from the host operating system (aside from the kernel which is ubiquitous of any OS level virtualization solution).

Another fundamental aspect of research reproduction is the preservation and validation of data, and Singularity has a feature under development that will ensure validation of container contents. Via direct integration of SHA256 hashing [47], Singularity will provide a method of container validation that guarantees that a container image being distributed has not been modified or changed. This is essential in ensuring compliance with White House Office of Management and Budget Circular A-110 [48], which states the need for data preservation and the ability to validate research results for federally funded projects. After the bootstrapping process, the SHA256 hash of the image file is generated and displayed for the user. When the image is later run using the − − *hash* option, its hash will be regenerated and displayed for the user to see.

When publishing scientific results, an author can distribute the Singularity image used in the paper alongside its hash, allowing others to independently validate the results using code that is verifiably identical to the code used in the original report. Any image repositories that build and serve Singularity images can also take advantage of this feature by storing the hash as metadata with the built images. Users that obtain images from these repositories can then use the hash to confirm a complete download of the image files.

#### User freedom

System integrators, administrators, and engineers spend a lot of time and effort maintaining the operating systems on the resources they are responsible for, and as a result tend to take a cautious approach on their systems. This leads to the common occurrence of mission-critical production systems being provisioned with old operating systems. These older operating systems may not receive critical security updates and also have fewer available software packages. It also leads to maintaining software or libraries that are either old or incompatible with the software that a particular user needs. Software building and installation is complex, and even with automation tools can be a non-trivial task due to incompatibilities or conflicts with other installed programs.

Singularity can give the user the freedom they need to install the applications, versions, and dependencies for their workflows without impacting the system in any way. Users can define their own working environment and literally copy that environment image (a single file) to a shared resource, and run their workflow inside that image.

#### Support on existing traditional HPC resources

There are a lot of container systems presently available [15, 49] which either are designed as enterprise solutions, a replacement for virtual machines, cloud-focused solutions, or they require kernel features that are not yet stable, not yet available, or both.

Replicating a virtual machine cloud-like environment within an existing HPC resource is not a reasonable task, but this is the direction one would need to take to integrate a technology like Docker into traditional HPC. The use cases do not overlap nicely, nor can the solutions be forcibly wed.

The goal of Singularity is to support existing and traditional HPC resources as easily as installing a single package onto the host operating system. For the administrators of the hosts, some configuration may be required via a single configuration file, however the default values are tuned to be generally applicable for shared environments.

Singularity can run on host Linux distributions from RHEL6 (RHEL5 for versions lower than 2.2) and similar vintages, and the contained images have been tested as far back as Linux 2.2 (approximately 14 years old). Singularity natively supports technologies such as InfiniBand and Lustre, while at the same time integrating seamlessly with any resource manager (e.g. SLURM, Torque, SGE, etc.) as a result of the fact that Singularity is run like any other command on the system. Singularity also includes a SLURM plugin [10, 50] that allows SLURM jobs to be natively run from within a Singularity container. The development and desire for this integration is a champion example of the strength of community developed, open-source software.

### Example use cases

A container solution that provides mobility of compute, reproducibility, user freedom, and extensibility across HPC resources is desired by many different user groups. Here we provide a sample of example use cases.

#### The academic researcher

The academic researcher wants to develop an analysis locally, meaning using a particular operating system, set of software, and library of functions, to work with some data to produce a particular output. The researcher then needs to be able to take that analysis, and move it to a different infrastructure to run at scale. The researcher then would like to publish and distribute the entire contained analysis and its corresponding hash alongside the results of the research, allowing others to easily reproduce and validate the results. Singularity is optimized for this use case. The academic researcher would make a Singularity container with scripts and dependencies, run it on his or her cluster, perhaps mapping drives to write data outputs, and then sharing the image and its hash with distribution of the work.

#### The server administrator

The server administrator is managing a shared, multi-tenant resource to a number of users. As it is a shared system, no users have root access and it is a controlled environment managed by the server administrator and his team. To keep the system secure, only this group is granted root access and control over the state of the operating system. If a user is able to escalate to root (even within a container) on the resource, the user can potentially do bad things to the network, cause denial of service to the host (as well as other hosts on the same network), and may have unrestricted access to file systems reachable by the container.

To mitigate security concerns like this, Singularity does not provide the ability to escalate permission inside a container. With Singularity containers, if a user does not have root access on the target system, the user cannot escalate privileges within the container to root either. The Server Administrator would instruct his users to have an endpoint (a local workstation, laptop, or server) where they have root access to perform write operations to images, and then move those images onto the cluster to run at scale. Considering the prevalence of laptops, especially at academic institutions, this is not an unreasonable or unmanageable mitigation.

#### Eliminate redundancy in container technology

A lot of time and energy have gone into developing Docker images, and Docker is being encouraged by several journals for reproducibility of tools [51]. Thus, a powerful use case is to run Docker images in an HPC environment using Singularity, which has been developed to work seamlessly with Docker. Image names that are local files can be swapped out for a docker endpoint, for example:

$ singularity shell ubuntu.img
$ singularity shell docker://ubuntu:latest

Would each create an interactive shell for an ubuntu image. The first is an image that the user has generated locally, and the second is creating an image on demand by downloading layers from the Docker Registry. This interchangeability works for bootstrap, import, and shell. Additionally, Singularity has functionality that enables users to bootstrap an image with a local Docker image that is not hosted on the Docker Registry.

#### Running a container at scale

The final example use case pertains to running a container at scale. The approach of using a single image for a container format is advantageous in that on a large, parallel file system, all metadata operations within the container occur within the container image as opposed to the metadata server.

## The singularity software

This paper encompasses a review of the software as of version 2.2. The initial version (1.0) was created by Gregory M. Kurtzer at Berkeley National Lab, and the author base has since expanded to include engineers from Stanford Research Computing (author VS), the University of Michigan (author MWB), along with substantial contributions from others (see Acknowledgements and the AUTHORS file within the distribution source code). This discussion of the software is catered to the user group, and for administration documents we point the reader to http://singularity.lbl.gov/admin-guide.

### The container

Singularity utilizes container images, which means that when a user enters and works within the Singularity container, he or she is physically located inside of this image. There is no standard definition for containers, and our usage of the term refers to a portable environment. For a detailed description of Linux Containers, which drive Docker and other container technologies, we refer the reader to Section 2 of Priedhorsky et. al. [30]. The Singularity container image encapsulates the operating system environment and all application dependencies necessary to run a defined workflow. If a container needs to be copied, this means physically copying the image. While a standard image file is typically used for containers, other container formats are also supported (Table 2).

**Table 2:** Container formats supported.

Format	Description
*directory*	Standard Unix directories containing a root container image
*tar*.*gz*	Zlib compressed tar archives
*tar*.*bz*2	Bzip2 compressed tar archives
*tar*	Uncompressed tar archives
*cpio*.*gz*	Zlib compressed CPIO archives
*cpio*	Uncompressed CPIO archives

In addition to the default Singularity container image, a standard file, Singularity supports numerous other formats described in the table. For each format (except directory) the suffix is necessary for Singularity to identify the image type

#### Supported URIs

Singularity also supports several different mechanisms for obtaining the images using a standard URI format:

- **http://**—Singularity will use Curl to download the image locally, and then run from the local image
- **https://**—Same as above using encryption
- **docker://**—Singularity can pull Docker images from a Docker registry, and will run them non-persistently (e.g. changes are not persisted as they can not be saved upstream)
- **shub://** Singularity can pull Singularity images from a Singularity image registry (Singularity Hub, under development)

#### Structure

A Singularity container has several files that directly interact with the container at runtime. These files and their descriptions are listed below:

1. /**singularity**—A file that contains a user-specified script to be run when the container is executed directly or through the ‘singularity run’ command.
2. /.**env**/ A directory that contains an arbitrary number of files to be sourced on container runtime. Files are to be named by the convention *XX* − *, where *XX* represents a two digit number describing priority (higher numbers have higher priority) and * represents an arbitrary string (e.g. 01 − *docker*)
3. **Entrypoint**—Scripts executed when corresponding singularity command is called, and passes the extra command line arguments into the executed binary
  - /.**exec**—Sources the environment files in /.env/ and executes the user specified command
  - /.**shell**—Sources the environment files in /.env/ and executes /bin/sh
  - /.**run**—Sources the environment files in /.env/ and executes the runscript located at /singularity
4. **Header**—Each singularity image contains an image header. The contents of this header are under development and will include:
  - SHA256 hash of the image (if generated).
  - Bit switch describing if the image has been modified since last singularity hash operation.
  - Bit switch describing if image is static (unmodifiable). Images with this bit set will be unable to be ran in writable mode.
  - Architecture of the image
  - Metadata associated with the image

#### Image contents

Within a particular container image one can include a base operating system’s application and library stack, custom or specific scientific programs, data, scripts and analysis pipelines. The container images, including these contents, are highly portable between Linux distributions, as long as the binary format (e.g. ELF x86_64) is compatible. For example a Centos or Debian image can be executed on Mint or Slackware.

#### Image permissions

The treatment of Singularity images as standard files simplifies management and access controls to well known POSIX based file permission. If you either own a container image, or have read access to that container image, you can start a shell inside that image. If you wish to disable or limit access to a shared image, you simply change the permission ACLs to that file.

#### Security

Singularity does not provide a pathway for privilege escalation (which makes it truly applicable for multi-tenant shared scientific compute resources). This means that in the runtime environment, a user inside a Singularity container is the same user as outside the container. If a user wants to be root inside the container, they must first become root outside the container. Considering on most shared resources the user will not have root access means they will not have root access within their containers either. This simple concept thus defines the Singularity usage workflow.

For more technical details, we direct the reader to our administrative documentation (http://singularity.lbl.gov).

### Singularity usage workflows

The standard usage workflow for working with an image typically means the user develops it locally on his or her own resource (laptop, workstation, virtual machine, etc), optionally compresses it, and moves it to another filesystem that has Singularity installed to run. If the user does not have root on that system, he or she will not be able to make any changes to the image once on that system. However, the user will be able to use the container and access the data and files outside the container as easily as he or she would on the original system or virtual machine where the image was developed. Images can serve as stand-alone programs, and can be executed like any other program on the host.

**Fig 1:**
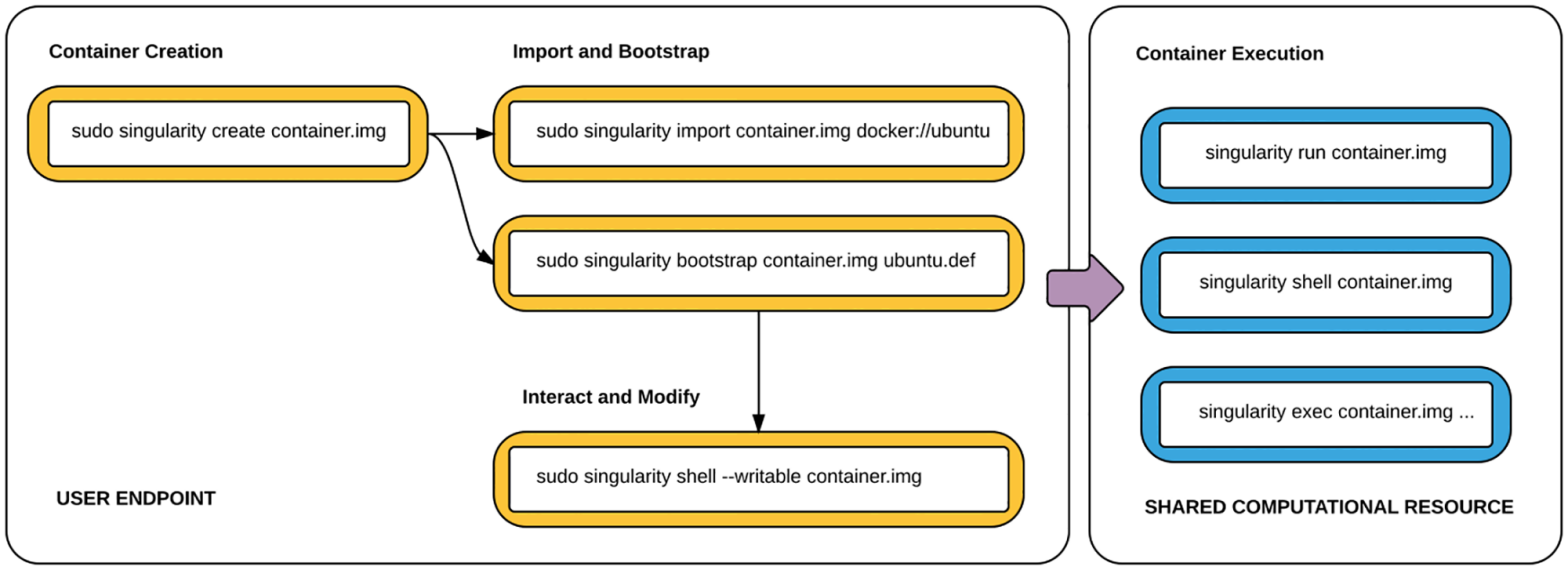
Singularity usage workflow. The standard Singularity Usage Workflow involves a working endpoint (left) where the user has root, and a container can be created, modified and updated, and then transferred to a shared computational resource (right) to be executed at scale.

The general workflow moves from development on an endpoint to a shared computational resource (Fig 1). One the left side, there is an endpoint that the user controls. This is typically a laptop, workstation, or server. In this space the user can create, modify, and update a container as needed. Once a container is created with the necessary applications, libraries and data inside, it can be easily shared to other hosts and executed without having root access. Making changes to the container again requires returning to the endpoint system with root, and re-uploading the container to the shared resource. In the case that a user does not have root on their endpoint (e.g., Windows), Singularity containers can be developed with a virtual machine, one that is provided at https://github.com/singularityware/singularity-vagrant.

### Docker integration

Singularity supports the ample amount of work that has gone into developing Docker images without relying directly on the user to install the Docker engine. This is done by way of harnessing the Docker Registry API, a RESTful interface that gives access to image manifests, each of which contains information about the image layers. Each layer is a compressed set of folders and files that can be extracted directly into a Singularity image. Additional information about the environment and runtime commands are also extracted from this manifest, and included into the Singularity image. By way of the docker uri (*docker*://) the user can bootstrap, run, and shell Docker images with only the requirement of an internet connection.

### Command line examples

Here we will provide some common Singularity use cases. A summary of commands is included in Table 3, and for more detailed description, we recommend the reader to see the User Guide at http://singularity.lbl.gov/user-guide. For each of these use cases, we have provided an example command using a local image (e.g., *ubuntu*.*img*) or an example image from the Docker Registry (eg, *docker*://*ubuntu*:*latest*).

**Table 3:** Singularity commands.

Global Options	
−*d* − −*debug*	Print debugging information
−*h* − −*help*	Display usage summary
−*q* − −*quiet*	Only print errors
− − *version*	Show application version
−*v* − −*verbose*	Increase verbosity +1
−*x* − −*sh* −*debug*	Print shell wrapper debugging information
**General Commands**	
*help*	Show additional help for a command
**Container Usage Commands**	
*exec*	Execute a command within container
*run*	Launch a runscript within container
*shell*	Run a Bourne shell within container
*test*	Execute any test code defined within container
**Container Management Commands (requires root)**	
*bootstrap*	Bootstrap a new Singularity image
*copy*	Copy files from your host into the container
*create*	Create a new container image
*export*	Export the contents of a container via a tar pipe
*import*	Import/add container contents via a tar pipe
*mount*	Mount a Singularity container image

Singularity command descriptions available via singularity –help. For all of the commands in the table, the general usage is: singularity [global options…] <command>[command options…] Options and arguments must be separated by spaces, and not equals (=) signs (e.g. –bind <path>).

#### Creating a container

A container is a file, and so creating it simply means creating the file, optionally with a particular size. In the example below, we create an image of size 4000MB.

$ sudo singularity create –size 4000 ubuntu.img

Once you have created the image file, it is essentially an empty bucket waiting to be filled. The process of filling the container is called “bootstrapping” and this is done by using a bootstrap definition recipe (e.g., ‘ubuntu.def’ in the example below) that installs a base operating system and then runs the commands necessary to install the application stack. The format for this command is as follows:

$ sudo singularity bootstrap ubuntu.img ubuntu.def

#### Shelling into a container

The user might want to launch an interactive shell within a container image. The following shows how the user would shell into a local container called ‘ubuntu.img’:

$ singularity shell ubuntu.img

If we add the − −*writable* or −*w* option we can write to the container, and we need root privileges:

$ sudo singularity shell –writable ubuntu.img

The user may also want to run the Singularity version of a Docker container on demand. The command below will use the Docker Registry to download image layers into a Singularity image to a temporary location, and then shell into it:

$ singularity shell docker://ubuntu:latest

#### Container run

For this next use case, a container acts as a function to perform a specific task. For example, a researcher might have a Python executable to take various inputs for an analysis. In this scenario, it is useful to know that each container can optionally have a runscript, a file called *singularity* at the root of the image file system. Given that this file exists, this script will be executed when the container is run as an executable. If no runscript exists, running the container as an executable shells into the image. The first way to run an image is to use the full command, “singularity run”, with any additional arguments:

$ singularity run analysis.img –input1 arg1 –input2 arg2

In the example above, the runscript is expected to know how to parse inputs and arguments. The second way to running a container is to treat the image as an executable:

$ ./analysis.img –input1 arg1 –input2 arg2

By default, Singularity launches the container image in read only mode (so it can be easily launched in parallel).

#### Container execute

We may be interested in sending a custom command to a container, in which case we are interested in *exec*. For example, here we use the executable *cat* to print the contents of a file to the terminal:

$ singularity exec centos.img cat /etc/redhat-release
CentOS Linux release 7.2.1511 (Core)

The command is run inside the container (and output printed to the local machine) without needing to shell into it, or touch the runscript. This would be useful for a quick glance at a file, or with the − − *writable* argument, to quickly make changes to the container image.

### Shared and mounted volumes

Singularity “swaps” out the currently running root operating system on the host for what is inside the container, and in doing so none of the host file systems are accessible once inside the container. As a workaround for this, Singularity will bind those paths back in via two primary methods: system defined bind points and conditional user defined bind points.

#### System defined bind points

The system administrator has the ability to define what bind points will be included automatically inside each container. The bind paths are locations on the host’s root file system that should also be visible within the container. Some of the bind paths are automatically derived (e.g. a user’s home directory) and some are statically defined (e.g. “bind path =” in /*etc*/*singularity*/*singularity*.*conf*).

#### User defined bind points

If the system administrator has enabled user control of binds (via “user bind control = yes” in this same configuration file, the user will be able to request bind points within container processes. The most typical example of this is the − − *bind* option. For example, here we bind /*tmp* to /*scratch*:

$ singularity run –bind /tmp:/scratch/ analysis.img

For more details on bind points, we direct the reader to http://singularity.lbl.gov/docs-mount.

### Compatibility with standard work-flows, pipes and IO

Singularity is a command line driven interface that is designed to interact with containers and applications inside the container in as transparent a manner as possible. This means that the user can not only run programs inside a container as if they were on the host directly, but also redirect IO, pipes, arguments, files, shell redirects and sockets directly to the applications inside the container. Here are some examples of this functionality:

1] singularity exec centos.img xterm
2] singularity exec centos.img python script.py
3] singularity exec centos.img python </path/to/python/script.py

In 1], we run the image’s xterm. In 2], we run script.py on our local machine using the python inside the container. In 3], we accomplish the same thing, but by way of a pipe.

### Bootstrapping containers

The idea of “bootstrapping” an image means starting from a previously generated template. In the case of Singularity, this means that we can bootstrap an image from scratch (building from a compatible host), or bootstrap a Docker image. Bootstrapping will be discussed in detail, as it is one of the more common use cases. For each bootstrap example that will be discussed, we can bootstrap from the command line, or use a build specification file, a file with a standard name “Singularity” (akin to “Dockerfile”) for more customization.

#### The bootstrap specification header

The Header describes the core operating system to bootstrap within the container. Here the user configures the base operating system features that are needed within the container. Examples of this include Linux distribution, docker image, version, and core packages. The **Bootstrap** keyword identifies the Singularity module that will be used for building the core components of the operating system. At the time of this writing, supported modules include yum, debootstrap, arch, and docker. For a complete overview see http://singularity.lbl.gov/bootstrap-image

#### Bootstrap specification sections

The rest of the definition is comprised of sections or blobs of data. Each section is defined by a **%** character followed by the name of the particular section. All sections are optional, and including one for setup **(%setup)**, post installation commands **(%post)**, specification of runscript commands **(%runscript)** and image testing **(%test)**. These sections are integrated during the bootstrap process in this order.

- **%setup**: This section is a script that will be executed on the host outside the container during bootstrap. The path to the container is accessible from within the running environment via the variable $*SINGULARITY*_*ROOTFS*.
- **%post** This section is also executed once during bootstrapping, but instead is run from inside the container. This is where the user should put additional installation commands, downloads, and configuration for the container.
- **%runscript**: This section is another script, but it does not get executed during bootstrapping. Instead it gets persisted within the container to a file called /*singularity* that is executed when the container image is run (either via the “singularity run” command or via executing the container directly). This is the file that, given a bootstrap of a Docker image, will run the same command specified by the Docker *ENTRYPOINT*. The user can change this default behavior to use *CMD* with a specification in the header.
- **%test**: This section is optional, and is run at the very end of the boostrapping process to give the user a chance to validate the container during the bootstrap. The user can also execute this script through the container itself, meaning that the container’s validity can always be tested as it is transported to different hosts.

#### Bootstrap example

The final bootstrap file might look like this, in the case of a Docker bootstrap:

BootStrap: docker
From: ubuntu:latest

%runscript
exec echo “This is what happens when you run the container” “$@”

%post
echo “Install additional software here”

For more information on bootstrapping images, including Docker and bootstrapping from scratch, we point the reader to our Bootstrap documentation (http://singularity.lbl.gov/bootstrap-image).

#### Best practices for bootstrapping

When bootstrapping a container, it is best to consider the following:

- Install packages, programs, data, and files into operating system locations (e.g. not /*home*, /*tmp*, or any other directories that might get commonly binded on).
- If any special environment variables need to be defined, add them to the /*environment* file inside the container. Files should never be owned by actual users, they should always be owned by a system account (UID <500).
- Ensure that the container’s /*etc*/*passwd*, /*etc*/*group*, /*etc*/*shadow*, and no other sensitive files have anything but the bare essentials within them.
- Do all bootstrapping via a definition file instead of manipulating the containers by hand (with the − − *writable* options), this ensures greatest possibility of reproducibility and mitigates the “black box effect”.

### Demonstrated validity

The utility of Singularity, and its demonstrated need and value to the scientific community, is best demonstrated by its speed of adoption. As of the time of this publication, Singularity is available on 36 research clusters, including national labs, companies, and major universities across several countries (Table 4). Validation of the software comes from the strongest test that is possible—centers across the nation with security experts have reviewed it and decided to install it for their users. For example, as of February 27th, already 1 million jobs using Singularity containers have been run on the Open Science Grid, with a projection that in several months, a milestone of 1 million per day [52].

**Table 4:** Singularity reported usage.

Site or Organization	System Name	Size (cores)	Purpose of the System
CSIRO	bragg-gpu	2048	broad base scientific
Genentech, Inc.			research
Georgia State University	Orion	362	research
GSI Helmholtz Center	Greencube	300,000	heavy ion physics
Holland Computing Center	Crane and Tusker	14,000	campus cluster
HPC-UGent	golett	2500	general scientific research
LBNL**	Lawrencium	30,000	general scientific research
Lunarc	Aurora	360	Research
McGill HPC Centre	guillimin	22300	Compute Canada cluster
Microway	Microway Research Cluster	192	ccientific benchmarking
MIT	openmind	1,176	neuroscience
NIH***	Biowulf	54,000	general biomedical research
Purdue University	Rice	11520	campus HPC resource
Purdue University	Conte	78880	campus HPC resource
Purdue University	Snyder	2220	campus HPC resource
Purdue University	Hammer	3960	campus HPC resource
Purdue University	Carter	10560	campus HPC resource
R Systems NA, Inc.	Oak1	1024	shared resource
R Systems NA, Inc.	Oak2	2048	shared resource
R Systems NA, Inc.	HOU1	5376	shared resource
Rutgers University	sirius	32	scientific SMP machine
SDSC*	Gordon	16384	cluster for XSEDE
SDSC*	Comet	47776	cluster for XSEDE
Stanford University	sherlock	12764	compute for Stanford
Stanford University	scg4	3920	genomics at Stanford
TACC****	Stampede	102400	NSF key resource, all fields
UFIT	HiPerGator	51,000	research computing cluster
Ulm University, Germany	JUSTUS	550	computational chemistry
UNF	Stark	64	fMRI analysis of the brain
University of Arizona	Ocelote	10000	general research
University of Arizona	ElGato	2300	GPU cluster
UNC Berkeley	Savio	7820	HPC for research
University of Chicago	midway.rcc.uchicago.edu	24196	university cluster
University of Leeds	MARC1	1236	bioinformatics, analytics
University of Manitoba	Grex	3840	generalHPC cluster
WU in St. Louis		2000	general cluster

HPC Clusters Using Singularity: At the time of this writing, this table shows the site or organization name, the system name, the number of cores, and the purpose for 36 clusters that have (reported) Singularity installed.

*San Diego Supercomputer Center

**Lawrence Berkeley National Laboratory

***National Institute of Health

****Texas Advanced Computing Center

The administrators of many of these major centers have chosen to provide Singularity to their researchers over the other options described earlier, and it is notable that this list represents reported usage, which is likely a subset of actual usage. Additionally, Singularity is providing containers for enterprise and production level environments at the San Diego Supercomputing Center [53], the Texas Advanced Computing Center [54], and the GSI Helmholtz Center for Heavy Ion Research [55]. Singularity has been featured on several well known news outlets in the HPC community [56–58], won an Editor’s Choice Award from HPCWire at the Supercomputing 2016 conference [59], and despite only being available for about six months, already has researchers publishing analyses using it [23].

### Future enhancements

Under development is a registry for images, Singularity Hub, which will provide users with an online web interface to connect Github repositories with the application and automatically build images in the cloud on pushes to the repositories. The images will then be available programmatically for running or other use on a local machine, cluster environment, or cloud resource. The availability of this resource will mean that an entire workflow, from image generation to running at scale, is possible without needing any environment with root privileges. Although Singularity Hub is still in the final stages of development, its core functionality is finished, and an additional URI is provided as an endpoint to Singularity Hub:

**shub://** Singularity uses the Singularity Hub API to download the image locally.

Singularity Hub is the connector that will make running Singularity containers across different local, HPC, and cloud environments automatic, and easy. Singularity Hub will allow for a common method to drive a thorough, computational review of Singularity’s operations. Thus, while this paper is scoped to be a descriptive summary to officially announce and describe the software, the reader should expect this comparison with the release of Singularity Hub.

## Conclusion

We have detailed the rationale for, software, and usage for Singularity containers, and invite the reader to visit http://singularity.lbl.gov for installation instructions across specific platforms, administrative documentation, complete command line usage, along with the latest information, documentation, support, and news.

### Contribution

Contributions are encouraged from users alike to improve the Singularity software. Specifically, desired features would be addition of more plugins for cluster or environment specific needs, further integration with other container software, and continued feedback from users and developers alike. We would like to thank the large group of users, and developers for all contributions to the software.
